# Role of the gut-brain axis in HIV and drug abuse-mediated neuroinflammation

**DOI:** 10.3389/adar.2023.11092

**Published:** 2023-03-03

**Authors:** Sudipta Ray, Susmita Sil, Muthukumar Kannan, Palsamy Periyasamy, Shilpa Buch

**Affiliations:** Department of Pharmacology and Experimental Neuroscience, University of Nebraska Medical Center, Omaha, NE, United States

**Keywords:** microbiome, drug abuse, neuroinflammation, HIV-1, gut-brain axis

## Abstract

Drug abuse and related disorders are a global public health crisis affecting millions, but to date, limited treatment options are available. Abused drugs include but are not limited to opioids, cocaine, nicotine, methamphetamine, and alcohol. Drug abuse and human immunodeficiency virus-1/acquired immune deficiency syndrome (HIV-1/AIDS) are inextricably linked. Extensive research has been done to understand the effect of prolonged drug use on neuronal signaling networks and gut microbiota. Recently, there has been rising interest in exploring the interactions between the central nervous system and the gut microbiome. This review summarizes the existing research that points toward the potential role of the gut microbiome in the pathogenesis of HIV-1-linked drug abuse and subsequent neuroinflammation and neurodegenerative disorders. Preclinical data about gut dysbiosis as a consequence of drug abuse in the context of HIV-1 has been discussed in detail, along with its implications in various neurodegenerative disorders. Understanding this interplay will help elucidate the etiology and progression of drug abuse-induced neurodegenerative disorders. This will consequently be beneficial in developing possible interventions and therapeutic options for these drug abuse-related disorders.

## Introduction

**FIGURE 1:**
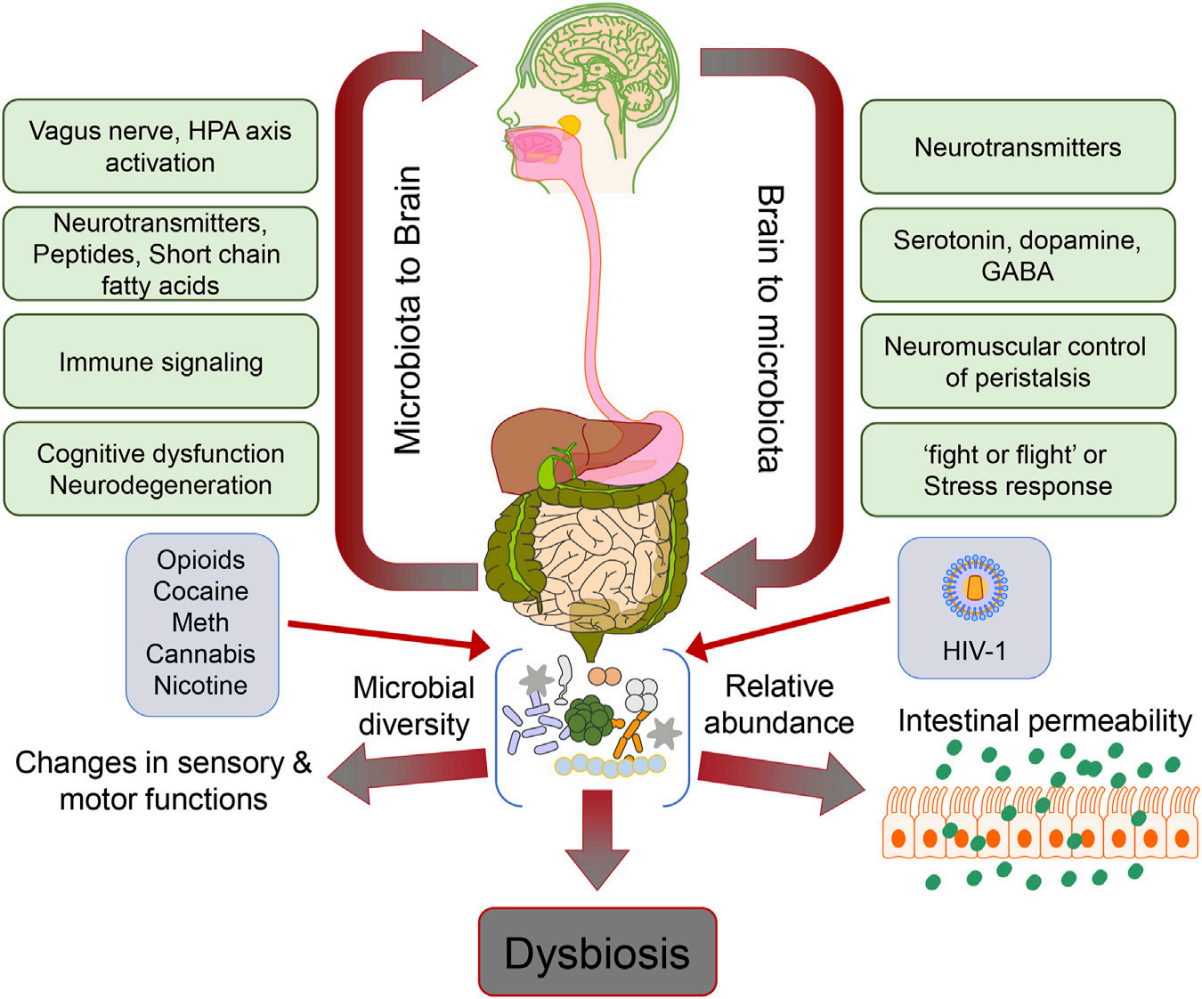
Schematic depicting the gut microbiome dysbiosis and its effects on the gut-brain axis in the context of HIV-1 and drug abuse.

Drug abuse is a significant global problem prevalent in those infected with Human Immunodeficiency Virus-1 (HIV-1). The most commonly abused drugs in HIV-1 infected individuals are opioids, alcohol, cocaine, cannabis, methamphetamine (Meth), and nicotine. Among all the drugs used, opioid abuse is a growing problem since opioids are often the mainstay of pain management in infected individuals. While these drugs effectively control the pain associated with HIV-1, their long-term use is associated with addiction, tolerance, and neurocognitive impairment, adding to the burden of behavioral deficits in HIV-1-infected individuals. When HIV-1-affected individuals use morphine, it may cause a loss of functional connectivity between the amygdala and the frontal cortex of the brain, insula, and striatum leading to neurodegenerative effects (1). Alcohol consumption in the form of ethanol is both toxic and has metabolic and addictive effects on the brain, accumulating over time with age, dose, and duration of exposure. Severe debilitating diseases of the central nervous system (CNS) and the peripheral nervous system are known to manifest due to alcohol consumption. For example, it is well established that prenatal alcohol exposure paves the way for lifelong behavioral, cognitive, and psychological problems, which account for a range of cognitive dysfunctions referred to as fetal alcohol spectrum disorders (2). Prolonged heavy alcohol abuse has been shown to lead to neurodegeneration and proportionate loss of cerebral white matter. The affected regions in chronic alcohol-related metabolic injury and degeneration include the cerebellum (especially the vermis), cortical-limbic circuits, skeletal muscle, and peripheral nerves (3). Specifically, alcohol impairs neuronal and glial cell functionality (3). Also, alcohol exerts prolonged effects at the cellular and systemic levels of the neurological networks, leading to neurodegeneration. Excess alcohol exposure is associated with specific diseases such as dementias, ataxias, and Niemann-Pick disease (4). Both excess and heavy alcohol consumption contribute to the development of neurodegenerative diseases, such as amyotrophic lateral sclerosis (ALS) and Alzheimer’s disease (AD). The brain is a major organ of alcohol accumulation, and this is linked to brain damage. Long-term alcohol abuse increases glutamate excitotoxicity and oxidative stress, resulting in neuronal damage (5). Besides alcohol, psychostimulants like cocaine, amphetamines, and nicotine have also been implicated in disruption of blood-brain-barrier (BBB), neural plasticity, and neuroinflammation (6, 7). There are case reports suggesting the association of cocaine overuse with accelerated neurodegeneration exhibiting symptoms similar to that found in Parkinson’s disease (8). It has also been shown that iron metabolism regulation and storage lead to dopamine accumulation in cocaine-abusing individuals, resulting in neuroadaptive changes in the basal ganglia (9). Other than genetic events, epigenetic events also play a major role in neurodegeneration mediated by abuse of substances such as cocaine and Meth, as well as opioids. Epigenetic changes are established by classical pathways, including the class III histone deacetylase-sirtuin family modifications by the stimulatory effects of drugs in the form of psychostimulants (10). Drugs of abuse have also been extensively reported to cause dysbiosis of the gut microbiome and, there is significant amount of evidence that links the dysbiotic gut microbiome to mental health and neurodegeneration (11-14). In this review, we summarize existing research, including preclinical and clinical studies about correlation between HIV-1-linked drug abuse and the intestinal microbiota, and the potential role of the resultant dysbiotic gut microbiome in the pathogenesis of neurodegenerative disorders (Figure 1).

## Gut microbiome and the gut-brain axis

The gut microbiome comprises a highly diverse repertoire of trillions of microbes that dwell in the gut linings and has been identified as a marker associated with various disease conditions. There is a standard composition of microbes in the gut for the metabolism and energy assimilation of the body. Several environmental, nutritional and genetic factors influence the multiplication of gut microorganisms and their compositional modifications (15). The human gut microbiome consists of various microbes that are beneficial to the body for metabolism and involved in a communication pathway to the CNS *via* the bidirectional “microbiota-gut-brain axis,” which was initially termed the “gut-brain axis.” In the 1960s, the brain was thought to control gut function which served as the basis for coining the term “gut-brain axis.” Later, bidirectional interactions between the gut microbiota and the CNS was discovered and was defined as the “microbiota-gut-brain axis (16-19). Recent findings implicate the role of the gut-brain axis in regulating behavior and responses to drugs and, thus, underpinning its role in reward and satiety (20-22). The vagus nerve physically connects the gut and the brain through an interplay of neurotransmitters and metabolites (23-26). The existence of specific microbes in the gut is known to regulate both the immune system (27-30) and inflammation (31-34). Both preclinical and clinical studies have demonstrated a pivotal role of the gut microbiota in brain functioning (35), mood (36), and behavior (37, 38). Gut microbiota regulates the differentiation and function of immune cells of the intestine, periphery, and brain (39-41). Growing evidence also points to the critical role of the gut microbiota and the immune system in regulating the pathogenesis of neurodevelopmental and neurodegenerative diseases (12).

There is an altered influx and efflux of microbial metabolites and immune mediators between the gut and brain, leading to impaired neurotransmission and the advent of many neuropsychiatric and neurological disorders (42). Microbiome changes, termed dysbiosis, result in acute and chronic stages of several diseases, such as depression (43). Further, dysfunctional glutamate neurotransmission is involved in the D-glutamate signaling pathway observed in AD models in which the gut microbiome metabolized D-glutamate influences the glutamate N-methyl-D-aspartate receptors and cognitive function in dementia patients (44). The gut microbiota has also been linked to the development of schizophrenia (42). The “gut-brain axis” encompasses several key signaling pathways. The immune system, the vagus nerve, or microbiota-modulating neuroactive compounds may drive these pathways. Existing literature also points toward the fact that bacteria present in the gut microbiome are responsible for the production and consumption of several mammalian neurotransmitters, such as dopamine, serotonin, norepinephrine, or gamma-aminobutyric acid (GABA). Reports suggest that, on the one hand, any change in the levels of these neurotransmitters by bacteria could impact host physiology, while on the other hand, any form of microbiota-based interventions could also alter neurotransmitter levels (45). A prime example of this regulation is the impact of the gut microbiome on tryptophan metabolism and the serotonergic system (46). In such a scenario, the interaction between gut microbes with drugs of abuse is complex since gut microbes can directly impact the response of an individual to a specific drug by enzymatically modifying the structure of the drug and, in turn, affecting its availability, activity, or toxicity in the system. The drugs of abuse can also influence the microbiome composition (47-51).

## Drug abuse and gut microbiome

Recent evidence implicates the gut-brain axis in the regulation of not only behavior but also a response to drugs in terms of reward and satiety. The vagus nerve connects the gut and brain, but several metabolites, hormones, and neurotransmitters regulate this connection. Such an influence of gut microbes on brain functions has been supported by studies in both preclinical and clinical models (52). During drug abuse, the gut-brain axis is disrupted, leading to modifications in the normal microbiota composition and dysregulated expression of neurotransmitters, bile acids, and metabolites, such as short-chain fatty acids (SCFA). Alterations in SCFA levels mediate tight junction dysfunction resulting in aberrant permeability of the gut epithelium, which can activate a wide range of proinflammatory signaling pathways (53). The hypothalamic-pituitary axis is linked to this inflammation in the gut, which subsequently sends feedback to the CNS, resulting in pain, stress, and anxiety (52). Herein, we discuss the role of the microbiota-gut-brain communication in the context of drug abuse in people living with HIV-1 (PLWH).

## Opioids and HIV

Opioids comprise a large class of compounds with different mechanisms of action and include heroin, morphine, oxycodone, fentanyl, methadone, buprenorphine, and nalorphine, among several others (54). Opioid receptors are widely distributed in the central and peripheral nervous systems and the digestive tract (55, 56). Prescription opioid drugs are used to treat moderate to severe chronic pain. Recently, the use of various opioid drugs and their abuse, which can lead to tolerance and dependence, has become a severe public health issue (57). According to the Centers for Disease Control and Prevention, out of the 92000 people who died from a drug overdose in 2020, 75% were due to prescription or illicit opioid use (58)**.** Most studies on the gut-brain axis and opioid abuse are based on the exogenous opioid compound morphine. Severe constipation is a primary physiological manifestation of chronic morphine use and has been linked to disruption of the gut epithelium and microbial dysbiosis (59). Animal models commonly used to study the pathways involved in the interaction between the host-gut microbiome and opioid drugs involve rodents, particularly mice and rats, primarily for economic reasons. However, recent studies have also focused on non-human primates (NHPs) as they are both physiologically and genetically closer to humans (60). The major outcome of these studies is a gut microbial imbalance or dysbiosis due to opioid use. Preclinical animal studies show that morphine exposure increases the abundance of pathogenic bacteria (*Flavobacterium, Enterococcus, Fusobacterium, Sutterella, Clostridium, Rikenellaceae,* and *Ruminococcus*). Once tolerance is developed, it causes a significant decrease in the quantities of beneficial bacteria (*Lactobacillus* and *Bifidobacterium*) (59, 61). It is difficult to extrapolate the data from the rodent preclinical models to the effect of morphine on human microbiota due to several factors such as genetic background, geographical setting, and lifestyle (60, 62). Human clinical studies also display variations in the presence of Bacteroidetes, Firmicutes, and Actinobacteria phylum of microbiota, consistent with rodent studies. However, there are only a limited number of studies utilizing NHPs to comment on any close association between the effect of opioids on gut microbiota in humans and that of NHPs.

It has been reported that opioid-induced gut dysbiosis, which causes structural changes in the gut epithelium, is responsible for tolerance and withdrawal behaviors. Disruption of the gut epithelium, in turn, allows bacteria and their toxic products to enter the host circulatory system, subsequently activating several inflammatory pathways and neuroinflammation. Withdrawal and tolerance linked to chronic opioid use have been related to this neuroinflammation (61, 63). The integrity of the gut epithelium depends on several factors, like the disruption of tigh-junction (TJ) organization and the restoration of the depleted epithelial layer by intestinal stem cells. The toll-like receptor (TLR) signaling is responsible for regulating intestinal TJ protein (TJP) organization. It has also been reported that morphine can disrupt the arrangement of the TJPs *via* modulation of myosin light chain kinase signaling (MLCK) in a TLR-dependent manner (64).

Opioid-induced microbial dysbiosis is responsible for continuous immune activation leading to HIV-1 disease progression. Several studies report that opioid addicts are at a greater risk of HIV-1 infection (65). Several factors, including the usage of contaminated needles and the nutritional status of the infected individual, could likely play a role in the heightened susceptibility of opioid abusers to HIV-1 infection. However, reports indicate that opioid use alone can also increase the risk of HIV-1 infection (66). There is ample evidence suggesting that HIV-1 infection disrupts the structure and function of the gut epithelium, leading to AIDS progression. Reports suggest that HIV-1 modulates tight junctions by disrupting CD4^+^ T cells, which are responsible for maintaining tight junctions (67). HIV-1 proteins such as Tat (transactivator of transcription) and gp120 have also been reported to disrupt tight junctions on epithelial cells in culture (68). Studies also report that simian immunodeficiency virus (SIV) infection results in early upregulation of proinflammatory cytokine IL-1β in the colon of the rhesus macaques (69) as well as in the intestine of HIV-1-infected patients (70), which, in turn, could activate the MLCK, resulting in mucosal damage. SIV-infected African green monkeys exhibit an accelerated depletion of CD4^+^ T cells in the intestine (71). An identical phenomenon is found in HIV-1-infected humans and SIV-infected rhesus macaques, suggesting that microbial translocation through the disrupted gut epithelium affects SIV disease progression.

Opioid users have been reported to display rapid HIV-1 disease progression while demonstrating severe long-term effects such as neurocognitive disorders (72). Certain opioid abusers infected with HIV-1 show elevated levels of lipopolysaccharide (LPS) in their serum compared to non-users, thus underscoring that disruption of the gut epithelium is more acute in HIV-1 patients who use opioid drugs (73). Preclinical and clinical studies done in HIV-1-infected patients indicate that morphine-mediated disruption of intestinal tight junctions involves activation of MLCK. This has also been validated in rodent models where combination of opioids and HIV-1 infection either synergistically and/or additively activate MLCK, leading to increased gut epithelium permeability, which is observed in HIV-1-infected patients misusing opioids (74). Opioids have also been reported to promote HIV-1 disease progression by disrupting the intestinal epithelial self-repair mechanism and reducing epithelial proliferation in bone marrow-liver-thymus humanized mice and in opioid-using HIV-1+ patients (75). Cumulatively these studies underscore the pivotal role of gut microbiota in the disease progression of HIV-1 infection while also demonstrating that opioid abuse by HIV-1 patients can lead to severe disruption of gut homeostasis, resulting in an accelerated progression of the disease in comparison to drug naïve, infected individuals.

## Cannabis and HIV

Despite controlling the HIV-1 viral load with combined antiretroviral therapy (cART), gut epithelium defects and intestinal CD4^+^ cell depletion continue to persist. In HIV-1 infected patients compromised gut barrier function is aided by the increase in apoptosis, and chronic inflammatory signals on the one hand and the decrease in proliferation and repair of epithelial cells, on the other hand. Alterations in tryptophan metabolism leading to defects in microbes that produce butyrate in PLWH and likely contribute to increased gut permeability have been reported (76-78). A dysfunctional gut epithelium allows inflammatory microbial products such as LPS in the periphery to be translocated (79-82). In particular, defects in the gut epithelium make HIV-1+ individuals vulnerable to increased exposure to proinflammatory ligands produced by gut microbiota (78, 83, 84). These alterations lead to poor HIV-1 disease outcomes, including associated neurocognitive disorders (77).

Cannabis effectively alleviates symptoms associated with HIV-1 disease and other conditions such as cancer and neuropathic pain (85). Cannabinoids act on inflammatory pathways through mechanisms distinct from agents such as non-steroidal anti-inflammatory drugs (NSAIDs) (86). Naturally occurring endocannabinoids, including cannabis, have antioxidative and anti-inflammatory characteristics that help in healing and restoration and thus can be used as adjunctive therapy. As a class, cannabinoids are generally free from the adverse effects of NSAIDs. A concise survey of the anti-inflammatory actions of the phytocannabinoids Δ^9^-tetrahydrocannabinol (THC), cannabidiol, cannabichromene, and cannabinol has been reported (85-90). Meta-analyses of several clinical trials have established the efficacy of cannabis in HIV-1-related neuropathic pain and nausea (85-92), although dosing and administration routes varied widely. Some studies suggest that titrating dosing to effectiveness and side effects is a valuable strategy for dose selection. While acute cannabis exposure disturbs cognition, how its long-term use affects brain function in the context of HIV-1 is yet to be elucidated clearly (93, 94). Medicinal use of cannabis is becoming rapidly accepted, and a state-level authorized disease management strategy (95, 96). Healthcare providers identify the potential benefits of cannabis by understanding the potential benefits of symptom management. However, a few clinical studies on patients using cannabis as therapy showed potential dependence or possible adverse effects (93, 97). A better understanding of the strategic use of cannabis could aid clinicians in better treatment and therapeutic options with their patients. Since not much research has been done to assess the effects of cannabis in PLWH, there is a dearth of reliable data for cannabis use recommendations in the clinical field.

The endocannabinoid system is a complex network of receptors and enzymes involved in synthesizing and detecting endogenous lipid ligands (98-100). Most human tissues express cannabinoid (type-1 and -2) receptors (98, 99). Cannabinoid receptors type-2 are densely expressed in diverse immune cell types, including macrophages, microglia, splenocytes, monocytes, and T-cells resident in the thymus, spleen, and bone marrow tonsils (98-100). Endocannabinoid system signaling pathways are essential in HIV-1 infection for several reasons and has been pursued as a target for future pharmacotherapy to reduce inflammation (98-100). In HIV-1 infection, cannabis use has been shown to reduce systemic inflammation and activate the immune system (101). Furthermore, HIV-1 DNA is reported to decline more rapidly in individuals taking antiretroviral therapy and using cannabis than those not using cannabis (102). Cannabis use in PLWH leads to aggravated dysbiosis and epithelial barrier dysfunction of the gut, along with chronic inflammation and consequential ill effect on overall health (79, 81, 82, 103). Chronic cannabis use is reported to lower the abundance of *Prevotella* and increase the abundance of *Bacteriodes* bacteria in the gut microbiome. Lower abundance of *Prevotella* leads to systemic mitochondrial dysfunction and reduction of gut SCFA production in cannabis users which is linked to impairment in cognitive function (104). It is also reported that administration of cannabidiol-rich cannabis extract resulted in increased abundance of A. *muciniphila* and significant decrease in *Alistipes finegoldii*, *Lachnoclostridium sp. YL32*, and *Ruminiclostridium sp. KB18* alongwith remarkable downregulation of mucin-2 which is associated with maintenance of gut integrity. The study also found upregulation of inflammatory markers IL-1β, CXCL1, and CXCL2 which points towards the disruptive effect of long-term cannabis use (105).

## Cocaine and HIV

Cocaine is one of the most commonly abused drugs among PLWH, and it has been suggested that it accelerates AIDS progression. Based on the evidence that the limbic system of the brain, comprising a set of interconnected regions regulating pleasure and motivation, is the primary site of action for cocaine helps explain its high potential for addiction and relapse. Cocaine, a commonly used psychostimulant among PLWH, is a cofactor for HIV-1 infection and progression to AIDS. Globally almost 22.5 million people worldwide are affected by cocaine use disorder, thus making it a significant public health crisis with a high socioeconomic burden (106). Although cocaine is known to have immunomodulatory functions (107-109), the underlying mechanism(s) by which cocaine accentuates HIV-1 replication remains unclear. There are reports that cocaine increases HIV-1 infection/replication by inhibiting HIV-1 protective chemokines and/or upregulating the HIV-1 entry co-receptor (110, 111). Cocaine is a potent vasoconstrictor and brain stimulant. Its abuse leads to severe neurological (fainting attacks, hemorrhagic brain strokes, CNS vasculitis, and encephalopathies), cardiovascular (cardiac arrhythmia and heart attacks), and gastrointestinal complications (112-117).

Cocaine abuse has been reported to alter the gut microbiota composition which in turn affects the uptake and clearence of neurotransmitters. One particular study reports higher accumulation of norepinephrine in intestines of cocaine-administred mice helped the resident *Citrobacter rodentium* to flourish which resulted in depletion of the intestinal neurotransmitter glycine. This also resulted in glycine depletion in circulation and cerebrospinal fluid of cocaine-administered mice, which was in correlation with increased hyperlocomotion and escalation of drug-seeking behavior (118). The authors also reported alteration of synaptic plasticity pathways at the transcriptome-level in the nucleus accumbens of the cocaine-administered mice, and also that the behavioral changes were reversed with dietary supplementation of glycine or sarcosine (118). Another study reports that cocaine administration in mice reduces the abundance of *Mucispirillum, Butrycicoccus, Ruminococcaceae, Pseudoflavonifractor, and Lachnospiracea* species of bacteria in the gut microbiota which are the involved in the synthesis of SCFAs involved in maintaining mucosal epithelium integrity. Cocaine administration resulted in alteration of TJPs of the gut membrane, upregulated expression of proinflammatory markers NF-ĸB and IL-1β, and also disruption of the mucosal permeability *via* MAPK/ERK1/2 signaling pathway (106). Also, in case of mice with reduced gut-bacteria, cocaine admistration resulted in increased sensitivity towards drug reward as well as increased locomotor-sensitivity (119). These studies reveal the critical role of gut microbiome in the behavioral effects of cocaine addiction. The research on the gut microbiome and its relationship with drug abuse is currently in its infancy with a bright future, and still a long way to go.

## Methamphetamine and HIV

Similar to other drugs of abuse, several preclinical and clinical studies have demonstrated that Meth induces alterations in the gut microbiome (49,120-123). However, there is a lack of evidence directly linking the gut microbiota with Meth-induced brain dysfunction (124). Meth has been reported to promote the release of norepinephrine and dopamine, leading to a markedly decreased intestinal contractility and motor capacity (125). This decrease in intestinal muscle tone is associated with oxidative and nitrosative stress, which, in turn, can cause neuronal injury and death in the intestine and disrupt intestinal barrier functioning (126). Disruption of the intestinal mucosal barrier increases the permeability of the gut epithelium and plays an essential role in contributing to anxiogenic behavior (127), stress (128), depression (129), cognitive decline (130), and eating and sleep disorders (131). Disruption of the intestinal barrier also leads to the leakage of several inflammatory factors (like TNF-α, interferon-γ, IL-6), microbes, and metabolites from the gut epithelium to the circulatory and lymphatic systems (132). It has been reported that Meth use can increase the permeability of the blood-brain barrier (133), thereby facilitating the entry of microbial communities and metabolites to enter the brain (134). In mouse models, Meth-exposure has been reported to increase the abundance of pathogenic bacteria in the fecal microbiota (120), with increased inflammation, reduced TJP expression in the intestine, and decreased relative quantity of probiotics and fecal metabolites. Further, Meth exposure was also shown to enhance the intestinal autophagy-associated flora, concomitantly leading to the induction of autophagy in the CNS (123). Intestinal inflammatory biomarkers, including the proinflammatory cytokines, are upregulated in Meth abusers and have been reported to infiltrate the brain regions related to depression (135), causing alterations in neurotransmitter metabolism, neuroendocrine function, and neuroplasticity. A recent study has also shown that gene sequencing of the 16S rRNA of the rectal swab samples collected from individuals using Meth, showed increased presence of bacterial species such as *Finegoldia, Peptoniphilus*, *Parvimonas,* and *Porphyromonas* and depletion of species like *Faecalibacterium* and *Butyricicoccus* (122). In line with this study, other studies have also shown that there were alterations in the composition of microbes present in the gut of Meth users with decrease in quantity of *Bacteroidaceae* and *Deltaproteobacteria,* and increased abundance of *Sphingomonadales*, *Xanthomonadales, Romboutsia* and *Lachnospiraceae* (49). Interestingly, these alterations have been reported in those bacterial species which had previously been demonstrated to be altered in individuals with psychotic syndromes, thus pointing towards a potential link between Meth abuse and psychotic disorders (49). Forouzan et al. showed that Meth exposure and withdrawal in rats resulted in gut dysbiosis, which was linked to depression-like behavior as evidenced by the forced swim test. However, the authors reported no alterations in anxiety-like behaviors which was assessed by either the elevated plus maze or the open field test (136).

HIV-1 has been reported to alter the human intestinal microbiome. An exciting study showed significant changes in the microbiome in the context of drug abuse and sexual behavior during HIV-1 infection. Rectal swab samples, urine drug test results, along with responses to substance use and sex behavior questionnaires were collected from 37 HIV-1-positive individuals at two-time points, in a 6-month gap period, in a group that was being evaluated for the effects of drug abuse in men who have sex with men. The samples were subjected to 16S ribosomal RNA gene sequencing, and the association of the data with behavioral factors was examined using 0-inflated negative binomial regression. Further analyses demonstrated that abuse of Meth and marijuana exhibit unique associations. Meth use was linked with increased *Granulicatella* and *Porphyromonas* organisms in HIV-1 patients and a decrease in abundance of *Collinsella*, *Ruminococcus*, and Parabacteroides organisms. In contrast, marijuana use was associated with an increased abundance of *Clostridium* cluster IV, Ruminococcus, *Fusobacterium*, and Solobacterium organisms and decreased *Acidaminococcus*, *Dialister*, *Prevotella*, *Anaerostipes*, and *Dorea* organisms. From this study, it can be concluded that drug use and sexual behavior are important factors associated with intestinal dysbiosis during chronic HIV-1 infection among young men who have sex with men (137). Further, studies are warranted in the field, specifically in association with HIV-1 infection and drug abuse-related disorders.

## Nicotine and HIV

Several reports have, on the other hand, demonstrated an association between nicotine and microbiome dysregulation (138-142)**.** In one study aimed at assessing the link between the smoking status of an individual and their intensity of smoking with the relative abundance of gut microbial species in 249 Bangladesh participants, it was reported that there was an increase in the relative abundance of *Erysipelotrichi* and *Catenibacterium* in current smokers in comparison to those who had never smoked (139). Another interesting study showed that long-term nicotine administration in rats resulted in alterations of gut microbiota, which was more prominent in rodents fed a high-fat diet than a regular chow diet, thus indicating diet-dependent changes (142). In line with this study, another study showed that cigarette smoke altered gut microbiota composition, which was linked to modifications in the distribution of primary bile acids and cholesterol homeostasis (138). Another study also showed that oral administration of nicotine in mice differentially reorganized the gut microbiome in a gender-specific manner and, furthermore, modified the levels of metabolites such as GABA and glutamate, which are involved in gut-brain communication (142). A recent study has also demonstrated that nicotine altered the gut microbiome and metabolites involved in the gut-brain axis in a sex-specific manner. This study employed high-throughput sequencing and gas chromatography-mass spectrometry to evaluate the effect of nicotine exposure on the gut microbiome and its metabolism in C57BL/6J mice in a sex-dependent manner, with special emphasis on the signaling pathways involved in the gut-brain axis. The 16S sequencing results from this study indicated that the composition of the gut microbiome was differentially altered by nicotine in both females and males. Also, the differential changes in the bacterial carbohydrate metabolic pathways were consistent with lower body weight gain in nicotine-administered males. Genes related to oxidative stress response and DNA repair were also explicitly upregulated in the gut microbiome of the nicotine-treated male mice. Analysis of the fecal metabolome demonstrated that several neurotransmitters, such as glutamate, GABA, and glycine, and neuroactive metabolites-leucine and uric acid, were also differentially altered in female *versus* male mice. This study showed a sex-dependent effect of nicotine on gut microbiome composition, functional bacterial genes, and the fecal metabolome (141). However, studies are lacking on gut-brain axis in the context of nicotine and HIV.

## Conclusion and future perspectives

Understanding the impact of the gut microbiome on gut-brain axis communication has been the topic of momentous research over the past few years. There is a mounting effort to delineate the mechanism(s) of this communication at all axis nodes. It has been now well-established that gut microbiota is crucial for the proper development and maintenance of brain functions. Additionally, as discussed above, there is accumulating evidence from preclinical and clinical studies that implicate the role of microbial dysbiosis in various psychiatric, neurological, and neurodegenerative diseases in the context of HIV-1 and drug abuse. However, it is still a very nascent field of research, and caution must be exerted in over-interpreting these studies. Many unanswered questions remain regarding the beneficial effects of probiotics, with extensive work required to test optimal dosing, strain, and timing in therapeutic applications. The emphasis in the field must shift from correlative analyses to prospective longitudinal study design, causative and mechanistic investigations, and larger-scale trials of potential therapeutic approaches, especially in the case of HIV-1 and drug abuse comorbidity. One big conundrum in microbiota-based research is the ideal definition of healthy microbiota. Inter-individual differences in the gut microbiota composition can be very critical, making it challenging to apply a “one size fits all” approach to target the microbiota. However, this also provides future opportunities for practical personalized medicine approaches. We have also moved from focusing on single bacterial strains as pathogens to an emphasis on nurturing an entire community of microbes, lest they become pathological entities. There are many challenges to conventional wisdom at play, with the possibility that the alterations in the gut microbiota noted in many CNS disorders may have a causal role in symptomatology and that many of the drugs used to treat those disorders could be toxic to or support the diversity of our gut microbes.
